# The biological significance of tumor grade, age, enhancement, and extent of resection in IDH-mutant gliomas: How should they inform treatment decisions in the era of IDH inhibitors?

**DOI:** 10.1093/neuonc/noae107

**Published:** 2024-06-24

**Authors:** Martin J van den Bent, Pim J French, Daniel Brat, Joerg C Tonn, Mehdi Touat, Benjamin M Ellingson, Robert J Young, Johan Pallud, Andreas von Deimling, Felix Sahm, Dominique Figarella Branger, Raymond Y Huang, Michael Weller, Ingo K Mellinghoff, Tim F Cloughsey, Jason T Huse, Kenneth Aldape, Guido Reifenberger, Gilbert Youssef, Philipp Karschnia, Houtan Noushmehr, Katherine B Peters, Francois Ducray, Matthias Preusser, Patrick Y Wen

**Affiliations:** Brain Tumor Center at Erasmus MC Cancer Institute, Rotterdam, The Netherlands; Brain Tumor Center at Erasmus MC Cancer Institute, Rotterdam, The Netherlands; Department of Pathology, Northwestern University Feinberg School of Medicine, Chicago, Illinois, USA; Department of Neurosurgery, Ludwig-Maximilians-University, Munich, Germany; German Cancer Consortium (DKTK), Partner Site Munich, Germany; Department of Neurology, Brigham and Women’s Hospital, Boston, Massachusetts, USA; Sorbonne Université, Inserm, CNRS, UMR S 1127, Institut du Cerveau, Paris Brain Institute, ICM, AP-HP, Hôpitaux Universitaires La Pitié Salpêtrière - Charles Foix, Service de Neurologie 2-Mazarin, Paris, France; UCLA Brain Tumor Imaging Laboratory, Department of Radiological Sciences, David Geffen School of Medicine at UCLA, Los Angeles, USA; Neuroradiology Service, Department of Radiology, Memorial Sloan Kettering Cancer, New York, New York, USA; Institute of Psychiatry and Neuroscience of Paris (IPNP), INSERM U1266, IMA-Brain, Université Paris Cité, Paris, France; Service de Neurochirurgie, GHU-Paris Psychiatrie et Neurosciences, Site Sainte Anne, Paris, France; Department of Neuropathology, University Hospital Medicine and CCU Neuropathology, German Consortium for Translational Cancer Research (DKTK), German Cancer Research Center (DKFZ), Heidelberg, Germany; Department of Neuropathology, University Hospital Medicine and CCU Neuropathology, German Consortium for Translational Cancer Research (DKTK), German Cancer Research Center (DKFZ), Heidelberg, Germany; DFB Aix-Marseille Univ, APHM, CNRS, INP, Inst Neurophysiopathol, CHU Timone, Service d’Anatomie Pathologique et de Neuropathologie, Marseille, France; Department of Radiology, Brigham and Women’s Hospital, Harvard Medical School, Boston, Massachusetts, USA; Department of Neurology & Brain Tumor Center, University Hospital Zurich & University of Zurich, Zurich, Switzerland; Department of Neurology, Memorial Sloan Kettering Cancer Center, New York, New York, USA; Department of Neurology, TC David Geffen School of Medicine at UCLA, Los Angeles, USA; Departments of Pathology and Translational Molecular Pathology, University of Texas MD Anderson Cancer Center, Houston, Texas, USA; Center for Cancer Research, National Cancer Institute, National Institutes of Health, Bethesda, Maryland, USA; Institute of Neuropathology, Medical Faculty, Heinrich Heine University and University Hospital Düsseldorf, and German Cancer Consortium (DKTK), Partner Site Essen/Düsseldorf, Düsseldorf, Germany; Center For Neuro-Oncology, Dana-Farber Cancer Institute and Harvard Medical School, Boston, Massachusetts, USA; German Cancer Consortium (DKTK), Partner Site Munich, Germany; Department of Neurosurgery, Ludwig-Maximilians-University, Munich, Germany; Department of Neurosurgery, Henry Ford Hospital+Michigan State University, Detroit, Michigan, USA; Department of Neurosurgery, Preston Robert Tisch Brain Tumor Center, Duke University, Durham, North Carolina, USA; Inserm U1052, CNRS UMR5286, Université Claude Bernard Lyon, Lyon, France; Hospices Civils de Lyon, Service de neuro-oncologie, LabEx Dev2CAN, Centre de Recherche en Cancérologie de Lyon, France; Department of Medicine I, Division of Oncology, Medical University of Vienna, Vienna, Austria; Center For Neuro-Oncology, Dana-Farber Cancer Institute and Harvard Medical School, Boston, Massachusetts, USA

**Keywords:** astrocytoma IDH-mutant, oligodendroglioma IDH-mutant and 1p/19q codeleted, prognosis, vorasidenib, WHO brain tumor classification

## Abstract

The 2016 and 2021 World Health Organization 2021 Classification of central nervous system tumors have resulted in a major improvement in the classification of isocitrate dehydrogenase (IDH)-mutant gliomas. With more effective treatments many patients experience prolonged survival. However, treatment guidelines are often still based on information from historical series comprising both patients with IDH wild-type and IDH-mutant tumors. They provide recommendations for radiotherapy and chemotherapy for so-called high-risk patients, usually based on residual tumor after surgery and age over 40. More up-to-date studies give a better insight into clinical, radiological, and molecular factors associated with the outcome of patients with IDH-mutant glioma. These insights should be used today for risk stratification and for treatment decisions. In many patients with IDH-mutant grades 2 and 3 glioma, if carefully monitored postponing radiotherapy and chemotherapy is safe, and will not jeopardize the overall outcome of patients. With the INDIGO trial showing patient benefit from the IDH inhibitor vorasidenib, there is a sizable population in which it seems reasonable to try this class of agents before recommending radio-chemotherapy with its delayed adverse event profile affecting quality of survival. Ongoing trials should help to further identify the patients that are benefiting from this treatment.

For the podcast associated with this article, please visit ‘https://soc-neuro-onc.libsyn.com/idh-mutant-glioma-and-idh-inhibitors’

**Key Points**

- The volume of the residual tumor of IDH-mutant tumors after surgery is of major prognostic significance for outcome, but not the age of the patient.
- IDHmt tumors that are recurring after radiotherapy and/or chemotherapy usually have novel genetic alterations which are associated with poor outcomes.
- An objective grading system of IDH-mutant gliomas based on molecular findings is needed.

The 2016 World Health Organization (WHO) classification of central nervous system (CNS) tumors that integrated isocitrate dehydrogenase (IDH) mutational status with histology in classifying gliomas, and the introduction of mutant IDH inhibitors (from here on: IDH inhibitors) into clinical practice has led to a reassessment of the biology and optimal therapies for grades 2 and 3 IDH-mutant gliomas. Under current guidelines, a “watch and wait” approach (ie, active monitoring without immediate adjuvant treatment after surgery) is typically restricted to patients with “low risk” IDH-mutant glioma. These are defined as younger patients (< 40 years) and after gross total resection or with limited residual disease (≤ 2 cm diameter after surgery), no functional deficits due to the tumor, and grade 2 histology.^1^ These recommendations are based on analyses from trials conducted prior to the discovery of the IDH mutation in 2008 and its introduction in the WHO classification of CNS tumors.^2,3^ Early trials on mutant IDH inhibitors suggested activity mainly in patients with non-enhancing IDHmt glioma tumors, as opposed to patients with enhancing tumors.^4–6^ Based on these observations, the recent phase 3 placebo-controlled INDIGO trial evaluated vorasidenib in a trial on IDH-mutant grade 2 glioma patients with measurable disease, who had undergone surgery as their only previous treatment, without an enhancing lesion on the MR scan and who were considered to be appropriate candidates for a watch and- wait approach.^7^ Benefit of vorasidenib was convincingly demonstrated: Median progression-free survival (PFS) improved from 11.1 months for patients in the placebo arm to 27.7 months for patients in the vorasidenib arm (HR 0.39, *P* < .001).^7^

A major question emerging from the INDIGO trial is whether these results are applicable only to patients fulfilling the narrow inclusion criteria of the trial, or whether current data on IDH-mutant glioma allow a broader biological perspective and allow generalizability beyond the INDIGO population (ie, beyond grade 2 and non-enhancing IDHmt tumors). To answer the question, a critical review is needed to evaluate if traditionally used risk factors for tumor progression and adverse outcomes truly capture the biology of the disease and correlate with outcomes of patients using the 2021 IDH-status-based glioma WHO CNS classification. This question is not only important in identifying patient groups that are most likely to benefit from treatment with IDH-mutant inhibitors; it has also major implications for patient referral for radiotherapy and chemotherapy after surgery.

## Clinical Risk Factors of IDH-Mutant Glioma

In the pre-WHO 2016 era, several prognostic factors were well established for outcome in low-grade glioma patients: Age, performance status, presentation with seizures versus presence of neurological deficits, size of the tumor, radiological characteristics including the presence of enhancement, tumor growth rate (TGR), tumor grade and treatment including extent of resection.^3,8,9^ The historical studies that identified these factors included both IDHmt and IDH wild-type (IDHwt) tumors, and it cannot be assumed that they remain valid for determining prognosis in IDH-mutant glioma patients following the introduction of the WHO 2016 classification.^10^ In that same period, (post hoc) analyses of the trials in grades 2 and 3 gliomas receiving adjuvant chemotherapy after radiotherapy showed clinical benefit mainly in patients with IDH-mutant gliomas, with much longer survival in patients with grade 3 IDHmt tumors compared to historical studies (Table 1).^2,11–15^ With the increasing use of early maximal safe surgery and radiotherapy followed by alkylating chemotherapy, the natural history of these tumors can no longer be studied. Moreover, long-held clinical assumptions such as the impact of age and tumor grade on the outcome of patients with diffuse glioma are challenged in the era of integrated histomolecular glioma classification based on IDH mutation and 1p/19q codeletion status.^16^ New, contemporary cohort studies on patients with IDH-mutant gliomas are needed on all aspects of diagnosis and treatment to understand the outcome of patients.

**Table 1.** The Range in Overall Survival in IDH-Mutant Glioma Grades 2 and 3 After Radiotherapy With Alkylating Chemotherapy as Reported in Several Larger Series (Source: Bell et al.,^12^ van den Bent et al.,^15^ Chang et al.,^14^ Lassman et al.,^11^ Minniti et al.^17^)

	Grade 2	Grade 3
Astrocytoma IDHmt	11.4–11.8 years	7.9–9.7 years
Oligodendroglioma IDHmt, 1p/19q codel	NR	13.2–14.2 years

NR, not reached.

## Clinical Factors Associated With Outcome of Patients With IDHmt Tumors

Several recent studies reported on the outcome of patients with IDH-mutant gliomas. Table 2 summarizes several studies that combined clinical factors with imaging, resection, or pathology data.^11,17–29^ Most focused on specific features (eg, associations with extent of resection, specific pathological and molecular findings, grade, and imaging) and did not co-analyze other important (clinical) variables. Another limitation of these studies is the uniformly retrospective design with highly heterogeneous treatment patterns, in particular, the variable use of chemotherapy or radiotherapy depending on perceived risk factors. Still, they provide useful information on the prognostic significance of clinical, tumor, and treatment factors in patients with IDH-mutant glioma.

**Table 2.** Overview of Clinical Series on IDH-Mutant Glioma and Identified Prognostic Factors, Including the Hazard Ratio for Overall Survival for Identified Significant Factors

Author	Tumor type, grade	*n*	Age	Performance status	grade	Preoperative Size/volume	Surgery/Postoperative size	Post-op treatment	location
Aoki^20^	OD 2,3	141	2.25 (0.89, 5.70)	NR	NS	NR	3.44 (1.59, 7.47) (Q)	NR	NS
	A 2,3	109	NS	NR	NS	NR	NS (*P* = .07) (Q)	NR	NS
Carstam^19^	OD2	126	1.05 (1.02, 1.08)	4.47 (1.70, 11.78)	NA	1.05 (1.02, 1.08)	NS (Q)	NS	NS
Carstam^18^	OD 2,3	79	NS	NS	NS	1.05 (1.01–1.09)	NS	NR	NS
	A 2, 3	89	NS	NS	NS	NS	1.02 (1.01–1.03)	NR	NS
Minniti^17^	A2	103	NS	NS	NA	NR	0.27 (0.08, 0.87) (E)*	NA	NS
Mair^21^	OD 2,3	183	NS	NS	NS	NR	NS (Q)	NS	NR
Pal’a^23^	OD, A 2	144	NS	NR	NA	NR	NS (Q)	20.2 (3.4–118.9)	NR
Dao Trong^23^	A, OD,2,3	167	NS	NS	NS	NS	NS (Q)	NS	NS
Tesileanu^24^	A3	432	2.30 (1.13 − 4.67)	1.49 (1.06 − 2.08)	NA	NR	1.77 (1.07 − 2.91) (Q)	NA	NR
Wijnenga^25^	A, OD 2	205	NS	NS	NA	1.01 (1.0–1.02)	1.70 (1.06–2.75) (E)	NS	NR
Park^26^	OD 2,3	86	1.07 (1.04–1.11)	NS	5.2 (1.7–15.3)	NR	NS (Q)	NS	NS
	A 2, 3	211	NS	0.97 (0.95–1.00)		NR	0.30 (0.16–0.59) (Q)	2.99 (1.51–5.93)	NS
EORTC 26951^11^	OD 3	80	0.36 (0.17, 0.69)	NS	NA	NR	NS (Q)	NA	NR
RTOG 9402^11^	OD 3	125	NS	0.36 (0.20, 0.64)	NA	NR	NR	NA	NR
Tran^27^	A2, 3	118	NS	NS	S	NS	1.02 (E)	NA	NR
Appay^28^	OD3,	483	4.0 (1.8–9.0)	NR	0.58	NR	0.58 (0.31–1.09) (Q)	NS	NR
	A3, 4	428	1.6 (1.1–2.5)	NR	2.9 (1.8–4.8)	NR	0.46 (0.28–0.75) (Q)	0.29 (0.19–0.46)	NR
Weller^29^	A2-4	258	NS	NS	3.1 (1.4-7.0)**	NR	2.61 (1.5-4.6) (Q)	NS	NR

Abbreviations: OD, oligodendroglioma; A, astrocytoma IDHmt, 2, grade 2; 3, grade 3; NA, not applicable; NS, not significant; NR, not reported.

Column surgery/postoperative size: (Q): qualitative = description extent of resection; (E): exact: measurement volume after resection; * Postoperative volume ≤ versus > 1 cm^3^ **grade 2 versus 3.

+ all treated with chemo-radiation, “study on elderly patients.

The prognostic effect of age was analyzed in a number of studies, with various age cutoffs. In most studies on patients with astrocytoma, IDHmt failed to identify age as a prognostic indicator.^17,27,29,30^ One study of grade 3 astrocytoma patients found an association only in patients over 60 years, while another study reported a modest association in patients over 50 years.^16,24^ In contrast, 3 studies on IDHmt and 1p/19q-codeleted oligodendroglioma found an association with age: Age over 60 (cutoff identified with regression analysis), age as a continuous variable, and age over 40.^11,19,20^ A study on grade 3 and 4 IDH-mutant glioma found a more pronounced association with age in grade 3 oligodendroglioma patients, using the observed median (49.5 years).^28^ Another study that investigated IDHmt and 1p/19q-codeleted oligodendroglioma patients over 60 years found no difference in comparison with patients under 60 years. In contrast, a report from the French POLA network showed worse outcomes in patients with IDHmt grades 3 and 4 tumors who were over 70 years old; most had been diagnosed with grade 3 IDH-mutant and 1p/19q-codeleted oligodendroglioma.^23,31^

Performance status was associated with outcome in some of the cohorts on IDHmt and 1p/19q-codeleted oligodendroglioma (either performance status or neurological function) but in only one study on grade 3 IDH-mutant astrocytoma.^11,19,24,26^ The French POLA network study on elderly patients reported significant associations with outcome and a variety of clinical factors basically reflecting neurological function.^31^

Most of these series incorporated either tumor volume, extent of resection, and/or postoperative volume, mostly using only descriptive, categorical methodology for the description of extent of resection (eg, biopsy versus resection or versus partial or complete resection) without an attempt to quantify the post-resection tumor volume. Still, the association of tumor size and type of surgery with the outcome of patients was noted in many studies and emerged as significant in multivariate analysis especially if a quantitative assessment of postoperative volume was part of the study.^17,18,25^ With respect to postoperative treatments, several papers reported unfavorable outcomes after adjuvant treatment (be it radiotherapy, chemotherapy, or both).^22,26^ Others found no association impact with outcome.^19,25^ However, invariably, these series reveal a bias towards the selection of postoperative treatment for patients with unfavorable risk factors (especially less than complete resections).^17,21,22,26,32,33^ A series on patients with grades 3 and 4 tumors reported improved outcomes if postoperative treatment had been given.^28^ Some studies are limited to patients having undergone radiotherapy with or without chemotherapy.^11,15,17^ Three prospective randomized studies found improved outcomes if chemotherapy was added to radiotherapy.^2,11,15^ Two studies addressed immediate postoperative treatment versus delayed treatment and found no effect on survival; but here also patient selection appears to have played a major role in the choice for early treatment.^17,21^ None of the studies that investigated tumor location reported an association with survival. Importantly, a series on conservatively managed resected grade 3 glioma observed a 3.4-year median interval between surgery and the next oncological treatment.^34^ Codeletion status, pre-and postoperative volume, and TGR were associated with the time to the next treatment.

## Association With Tumor Size, Extent of Resection and Outcome

Maximal safe microsurgical resection is the standard of care for patients with IDH-mutant gliomas with and without 1p/19q-codeletion and has been associated with improved outcome.^1^ In the absence of randomized studies, one might argue that extent of resection reflects an indirect marker for tumor localization, preoperative tumor size, or invasiveness all with an inherently worse prognosis. Preoperative tumor size may be prognostic for patient outcome as larger tumors may increase the probability of infiltration into eloquent areas, limit resectability, and signify a greater risk of malignant transformation.^18,25,32,35^ To assess the relationship between the extent of resection and patient outcome, recent studies on grade 2 IDH-mutant glioma patients focused on the quantification of residual tumor volume (measured in cm^3^) which came out of the analyses of higher relevance than the relative -percentage- of tumor volume reduction.^18,25,32,35–37^ These studies show that preoperative tumor size was prognostic in both IDHmt astrocytoma and IDHmt and 1p/19q-codeleted oligodendroglioma patients, whereas residual tumor volume was predominantly associated with prognosis in IDHmt astrocytoma patients. When astrocytoma IDH-mutant patients were stratified by residual tumor volume, survival curves of astrocytoma patients were split within the first 5 years following initial resection.^25,32,35^ In contrast, patients with IDH-mutant and 1p/19q-codeleted oligodendroglioma seem to derive a survival benefit from smaller postoperative tumor volumes only after more prolonged follow-up (>5–10 years).^25,32^ The role of supramaximal resection in IDH-mutant glioma remains controversial, due in part to the lack of a reliable method to quantify the extent of resection beyond the T2/FLAIR-hyperintense tumor borders. Functional and anatomical borders might be key to guide resection in selected cases but the patient benefit of further increasing the extent of resection will need to be weighed against the risk for neurologic deficits.^32,38–40^ While it remains unclear whether longer observation periods or larger series will reveal a trend towards a survival benefit after supramaximal resection of IDH-mutant and 1p/19q-codeleted oligodendroglioma, the yet considerable median follow-up over 11.7 years in a recent study supports the notion that the benefit of resection beyond the tumor borders is likely to be limited in patients with IDHmt and 1p/19q-codeleted oligodendroglioma.^32^ Studies on the prognostic role of extent of resection for grades 3 or 4 IDHmt astrocytomas and grade 3 IDHmt and. 1p/19q-codeleted oligodendroglioma patients are rare. More complete resection with lower residual T2-weighted tumor volumes was also associated with favorable survival in 113 patients with IDHmt astrocytomas, of which 86 patients had grade 3 histology (and the remaining 27 patients had grade 4 histology); this finding was confirmed in other retrospective studies on astrocytomas grade 3 and 4.^37,38,41^ A small single-institutional study on patients with grade 3 IDH-mutant and 1p/19q-codeleted oligodendroglioma failed to detect an association between the extent of resection and survival.^42^

## Imaging and Contrast Enhancement

### Relation Between Tumor Grade and Enhancement, Patient Outcome

Survival of patients with IDHmt glioma correlated with contrast enhancement on MR imaging in several series.^43–46^ In patients with IDHmt and 1p/19q-codeleted oligodendroglioma, a positive correlation has been reported between MR contrast enhancement and grade.^47,48^ Neo-angiogenesis and mitotic counts were independently associated with TGRs (TGR) ≥ 8 mm/year.^49^ Contrast enhancement in grade 3 IDH-mutant and 1p/19q-codeleted oligodendroglioma patients was associated with worse outcome, with larger tumor volumes and with several molecular factors.^50,51^ Contrast enhancement had a sensitivity of about 60% in identifying grade 3 IDH-mutant and 1p/19q-codeleted oligodendroglioma, but up to 50% of grade 2 IDHmt and 1p/19q-codeleted oligodendroglioma may show some enhancement and a substantial proportion of grade 3 IDHmt and 1p/19q-codeleted oligodendroglioma do not.^52,53^ In IDH-mutant astrocytoma, a positive association has also been reported between MR contrast enhancement and grade.^47,53–55^ Still, on initial brain MRI only 60% of grade 3 IDH-mutant astrocytomas showed patchy and faint enhancement, whereas 20% to 50% of grade 2 IDH-mutant astrocytomas showed some enhancement.^43,47^ Ring enhancement has been associated with grade 4 IDH-mutant astrocytomas, and marked enhancement was associated with the presence of homozygous *CDKN2A* deletion.^47,56^ However, the identification of homozygous *CDKN2A* deletion based on MRI had limited sensitivity (80%) and specificity (58%).^56^ Nodular and ring enhancement patterns on contrast-enhanced MRI have also been reported in grade 3 IDH-mutant astrocytomas, and were associated with worse outcomes.^43,47,51^ In previously non-enhancing IDH-mutant gliomas, contrast enhancement at progression typically indicates tumor progression to a higher grade of malignancy, associated with more aggressive behavior of the tumor and a worse prognosis.^57^ Both temozolomide and radiotherapy have been associated with the induction of novel genetic alterations (hypermutation, small and large DNA deletions) associated with poor outcome, implying a change in biology induced by oncolytic treatment.^57–59^ However, as a word of caution, studies have shown that 25%–30% of patients with IDH-mutant gliomas may develop pseudoprogression following radiotherapy.^60,61^

### TGR as a Measure of Prognosis

Quantitative longitudinal studies of imaging growth patterns in patients with IDH-mutant glioma have confirmed the continuous tumor growth without treatment and established correlations with molecular status.^49,62–64^ Growth was slower in IDH-mutant and 1p/19q-codeleted oligodendroglioma than in IDHmt astrocytomas, and was also slower in grade 2 IDH-mutant and 1p/19q-oligodendroglioma codeleted than in grade 3 oligodendroglioma, IDH-mutant and 1p/19q-codeleted.^49,62^ Similarly, TGR correlated with grade in IDH-mutant astrocytoma (grades 4 > 3 > 2). In a cohort of IDHmt glioma patients on active surveillance (*n* = 128) a continuous percentage tumor volume growth rate per 6 months of 10.46% (95% CI: [9.11%, 11.83%]) and a doubling time of 3.5 years (95% CI: [3.10–3.98]) was noted, with higher rates in the presence of homozygous CDKN2A deletion.^63^ Each tumor volume increase of one natural logarithm was associated with a more than 3-fold increase in risk of death. When quantifying the tumor growth by the evolution of the mean tumor diameter over time, a cutoff ≥ 8 mm/year was systematically associated with IDHmt and 1p/19q-codeleted oligodendroglioma grade 3 rather than grade 2, and with either IDHmt astrocytomas grade 3 or 4.^65^ Using the current WHO classification, spontaneous tumor growth was observed to be a predictor of tumor progression requiring further oncological treatment in oligodendrogliomas and being a predictor of time to malignant transformation and of overall survival of patients with IDH-mutant glioma.^49,62,63,65,66^

## Histologic Parameters for Distinguishing Grades 2 and 3 IDH-Mutant Gliomas

The traditional method for distinguishing histologic grade 2 from grade 3 in diffuse gliomas is based on the microscopic assessment of features of focal or dispersed anaplasia (such as increased cell density and nuclear atypia), and mitotic activity. This grading system is however subject to considerable interobserver variation.^67^

### Grading of IDH-Mutant Astrocytoma

The 2021 WHO classification of CNS tumors states that in contrast to IDH-mutant astrocytomas grade 2, IDHmt astrocytomas grade 3 “exhibit focal or dispersed anaplasia and display significant mitotic activity.”^68^ IDHmt astrocytoma grade 3 may also feature atypical mitoses and/or multinucleated tumor cells, but microvascular proliferation, necrosis, and homozygous *CDKN2A/CDKN2B* deletion, ie, criteria for CNS WHO grade 4, are absent.^68^ Studies performed in the pre-IDH era indicated that diffuse astrocytomas with ≥ 2 mitoses per 10 high power fields (HPF) were associated with shorter survival than those with 0 or 1 mitoses and this threshold has been used by neuropathologists for the designation of WHO grade 3.^69,70^ In several recent studies of IDH-mutant astrocytoma cohorts, these thresholds for mitotic activity were not corroborated and no difference in survival between grades 2 and 3 tumors were observed.^30,71–73^ However, others have demonstrated that WHO grading schemes can stratify risk among patients with grades 2–4 IDH-mutant astrocytomas, yet with opportunity for improvement.^29,74–77^ A study based on selected patient cohorts included in EORTC trials 26053 (CATNON) and 22033-26033 reported that ≤ 2 mitoses per 10 microscopic HPF was significantly associated with longer PFS in patients with IDHmt astrocytoma without homozygous *CDKN2A/CDKN2B* deletion.^78^ A population-based report on clinical outcomes of IDHmt astrocytoma patients who were diagnosed according to the 2016 WHO classification of CNS tumors demonstrated that patients with WHO grade 2 IDH-mutant astrocytomas had a modest, but statistically significant, higher survival rate at 1 year than patients with IDH-mutant grade 3 astrocytomas (97.9% and 94.4%).^79^ A study of 118 IDH-mutant astrocytoma patients demonstrated that mitotic count (≥ 6/3mm^2^) was associated with a shorter time to postoperative treatment.^27^ Patients with tumors with mitotic activity lower than the cutoff and a post-surgical residual volume < 1 cm^3^ appeared to be the optimal candidates for observational follow-up. Studies of the proliferative index (eg, based on Ki-67 immunostaining) have not identified a robust cutoff to distinguish grade 2 from grade 3 IDHmt astrocytomas, although several studies reported worse outcomes in patients whose tumors displayed high proliferative activity.^27,71^

### Grading of Oligodendroglioma IDHmt and 1p/19q Codeleted

Similar to IDHmt astrocytomas, IDHmt, and 1p/19q-codeleted oligodendroglioma represent a continuous spectrum ranging from indolent and well-differentiated tumors to those that are rapidly progressing. Here the grading scheme is also based on morphologic features. The histological criteria for IDHmt and 1p/19q-codeleted oligodendroglioma grading were established in the pre-IDH era and have been maintained in the 2021 WHO classification of CNS tumors. These criteria were largely based on work which indicated that grade 3 tumors should be distinguished from grade 2 tumors by either the presence of brisk mitotic activity (≥ 6 mitoses per HPF; ≥ 2.5 mitoses/mm^2^), microvascular proliferation, or necrosis.^80^ However, the 2021 WHO CNS classification emphasized that “data defining a clear cutoff point for a mitotic count that distinguish CNS WHO grade 2 from CNS WHO grade 3 of IDHmt 1p/19q co-deleted oligodendroglioma are not available.”^68^ Multiple studies demonstrated that patients with grade 2 IDHmt and 1p/19q-codeleted oligodendroglioma have significantly longer survival compared to patients with grade 3 tumors based on the above histologic criteria^30,74,76^ However, recent studies of IDHmt- and 1p/19q-codeleted oligodendroglioma grade 3 indicate that patients with tumors displaying elevated mitotic activity (≥6/10 HPF; 0.24 mm^2^) but no microvascular proliferation or necrosis have significantly longer PFS and OS than those whose tumors show microvascular proliferation or necrosis.^81,82^ This suggests that elevated mitotic activity alone is not a strong prognostic marker for aggressive clinical behavior in this tumor type. The variability noted in study outcomes based on mitotic thresholds may be due to an inherent lack of reproducibility in the microscopic assessment of mitotic count, reflecting challenges in consistently recognizing mitoses, in the variable selection of microscopic fields, and in non-standardized counting techniques.

## Prognostic Genetic Markers

With the recent advances in molecular diagnostics, an important clinical question is whether molecular markers can provide a better way of risk stratification for patients with IDH-mutant glioma. More potential markers have been identified in IDH-mutant astrocytoma than in IDH-mutant and 1p/19q codeleted oligodendroglioma patients.

## Prognostic Genetic Markers: IDHmt Astrocytomas

Non-canonical IDH mutations have been associated with a better outcome in astrocytoma, possibly related to different levels of 2-HG production.^83^

### CDKN2A/B Hemizygous Deletion and Mutation

*CDKN2A*/B homozygous deletion is now recognized as a criterion for establishing the diagnosis of IDH-mutant astrocytoma grade 4, based on the finding of short overall survival associated with loss of both alleles.^75,84–86^ However, the presence of *hemizygous* deletion of *CDKN2A/B* was also shown to be a marker of less favorable outcome prognosis in patients with IDH-mutant astrocytomas when compared to patients with *CDKN2A/B* non-deleted tumors. Recent investigations found a significantly shorter survival among patients with IDHmt astrocytomas (all grades included) with hemizygous deletion of *CDKN2A/B* on multivariate analysis in independent datasets, with an intermediate overall survival of patients with hemizygous deletion of CDKN2A/B compared to patients with *CDKN2A/B* homozygously deleted tumors and patients whose tumors lacked copy number losses of *CDKN2A/B*.^87,88^ Hemizygous deletion was identified in 10% of grade 2, 27% of grade 3, and 33% of grade 4 IDHmt astrocytomas. The finding of any allelic loss of *CDKN2A/B* was associated with shorter survival on multivariate analysis, which included histologic grade. Two studies identified mutation of *CDKN2A/B,* an uncommon event in IDH-mutant astrocytoma (2.6%), to be associated with a poor prognosis similar to *CDKN2A/B* homozygous deletions on univariable analysis.^89^ The prognostic role of *CDKN2A/B* promoter methylation remains unclear.

### Alteration of Other RB Pathway Genes

**Figure 1.**
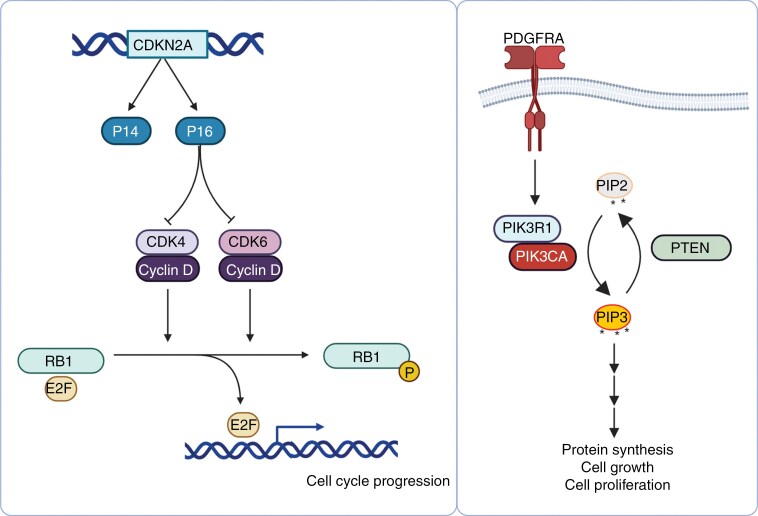
Schematic representation of the retinoblastoma (Rb) and receptor tyrosine kinase (RTK) pathway.

Analyses of large cohorts of patients with IDH-mutant astrocytoma grades 2–4 have shown that *CDK4* amplification is associated with shorter survival on multivariate analysis.^88,90^ Other studies have concluded that *CDK4* amplification, when considered by itself, was not associated with poor prognosis on univariate analysis.^75,91^ Since *CDK4* is a member of the RB pathway (Figure 1) and its amplification is mutually exclusive with *CDKN2A/B* homozygous deletion and *RB1* mutation, some investigators have explored alterations of RB pathway members as a single risk factor. Multivariate analysis of sizable patient cohorts reported that when altered RB pathway genes (*CDKN2A/B* homozygous deletion, *CDK4* amplification, or *RB1* mutation) were considered together, it was a strong and statistically significant predictor of poor prognosis among patients with grade 2 or 3 IDHmt astrocytoma.^92,93^ Others have used this approach to combine *CDK4* amplification and *CDKN2A/B* homozygous as a single risk variable and demonstrated a significant association with shorter overall survival when either one of these findings was present.^77^ Similarly, a copy number analysis model for predicting outcomes in histologic grades 2 and 3 IDHmt astrocytomas indicated that the presence of either homozygous *CDKN2A/B* homozygous deletion or *CDK4* amplification was associated with shorter survival and that the additional finding of chromosome 14 loss predicted an even shorter survival.^76,94^ Homozygous deletion of *RB1* was strongly associated with inferior overall survival among IDH-mutant astrocytomas on univariate analysis.^75^ Analysis of 2 cohorts of histologic grades 2–4 IDH-mutant astrocytomas have demonstrated shorter survival associated with *CCND2* amplification.^75,95^ Both mutual exclusivity with homozygous deletion of *CDKN2A* and the association with grade are suggestive of a role for *CDK6* amplification in tumor malignancy. Similar correlations with tumor grade are found for all members of the RB pathway mentioned in this paragraph (Table 3).

**Table 3.** The Frequency of Specific Alterations in Astrocytoma, IDHmt of Various Grades

gene	dataset	II	WHO2016 grade III	IV
CDKN2A	TCGA	4/111(4%)	7/104(7%)	4/16(25%)
	MSK	0/44 (0%)	13/92 (14%)	11/38 (29%)
	Korshunov			42/97 (43%)
	Lee	0/16 (0%)	0/43 (0%)	14/36 (39%)
CDK4	TCGA	1/111(1%)	5/104(5%)	5/16(31%)
	MSK	0/49 (0%)	5/100 (5%)	5/42 (12%)
	Korshunov			21/97 (22%)
	Wong			15/53(28%)
	Lee	0/16 (0%)	0/43 (0%)	5/36 (14%)
CDK6	TCGA	0/111(0%)	3/104(3%)	2/16(13%)
	MSK	1/49 (2%)	2/100 (2%)	1/42 (2%)
	Wong			5/53(9%)
	Lee	0/16 (0%)	1/43 (2%)	0/36 (0%)
RB1	TCGA	2/111(2%)	2/104(2%)	0/16(0%)
	MSK	0/49 (0%)	1/100 (1%)	4/42 (10%)
	Korshunov			11/97(11%)
PDGFRA	TCGA	1/111(1%)	9/104(9%)	1/16(6%)
	MSK	0/49 (0%)	8/100 (8%)	5/42 (12%)
	Korshunov			18/97 (19%)
	Lee	0/16 (0%)	0/43 (0%)	6/36 (17%)
PIK3CA	TCGA	2/111(2%)	1/104(1%)	1/16(6%)
	MSK	0/57 (0%)	8/117 (7%)	8/46 (17%)
	CGGA	1/20(5%)	0/22(0%)	2/13(15%)
	Korshunov			12/97(12%)
	Wong			2/53 (4%)
	Lee	0/16 (0%)	1/43 (2%)	3/36 (8%)
PIK3R1	TCGA	2/111(2%)	3/104(3%)	3/16(19%)
	MSK	0/57 (0%)	6/117 (5%)	5/46 (11%)
	CGGA	0/20(0%)	1/22 (5%)	3/13(23%)
	Korshunov			1/97(1%)
	Wong			3/53 (6%)
MYCN	TCGA	2/111(2%)	0/104(0%)	2/16(13%)
	MSK	1/49 (2%)	5/100 (5%)	5/42 (12%)
	Korshunov			12/97(12%)
	Wong			4/53 (8%)
	Lee	0/16 (0%)	0/43 (0%)	3/36 (8%)
CCND2	TCGA	9/111(8%)	8/104(8%)	3/16(19%)
	MSK	1/49 (2%)	4/100 (4%)	4/42 (10%)
	Korshunov			21/97 (22%)
	Lee	0/16 (0%)	0/43 (0%)	2/36 (6%)

Datasets: TCGA^95^; MSK^96^; CGGA^97^; Korshunov^98^; Wong^99^; Lee.^100^ Grading was extracted from the manuscripts/datasets and was done according to presented (WHO2016), except for Lee et al (WHO2021).

### Tyrosine Kinase Receptor/PI3K/PTEN Pathway

In several studies, PI3K pathway alterations (Figure 1), as defined by either *PIK3CA* or *PIK3R1* mutation were a marker of poor prognosis among patients with grades 2 and 3 IDH-mutant astrocytomas but not invariably.^90,92,93,100^ The positive correlation between tumor grade and mutation frequency in both *PIK3CA* and *PIK3R1* is also suggestive of a negative prognostic impact (Table 3).^96–99,101,102^ Multiple studies of large cohorts of patients with IDH-mutant astrocytomas have demonstrated that *PDGFRA* amplification is associated with poor prognosis, even in histological grade 2 tumors (Figure 1).^75,77,90,93^ The increased frequency of *PDGFRA* amplification in higher-grade tumors, and the high frequency in grade 4 IDH-mutant astrocytomas also points towards a negative prognostic impact (Table 3).

### MYCN Amplification

*MYCN* amplification has been shown to be associated with shorter survival in patients with IDH-mutant astrocytomas (grades 2–4)^75,93,96^ The frequency of *MYCN*-amplification increases with WHO 2016 tumor grade (Table 3), and was relatively high (8%–12%) in 2 separate cohorts of grade 4 IDHmt astrocytomas.^99,101^

### Chromosomal Instability and Tumor Mutational Burden in IDH-Mutant Astrocytoma

Increased aneupleudy and/or total CNV load is associated with poorer prognosis and poorer prognostic methylation classes in IDH-mutant astrocytomas.^24,73,75,103,104^ High mutational burden has been described in recurrent IDHmt astrocytoma in particular following treatment with temozolomide due to defects in mismatch repair genes, which is associated with enhancing recurrences and a worse prognosis and development of discontiguous disease.^57,105^

## Prognostic Genetic Markers: IDHmt and 1p/19q-codeleted Oligodendroglioma

### Chromosomal Arm 9p Loss and CDKN2A/B Homozygous Deletion

Deletions on 9p have been associated with histological grade 3 and contrast enhancement on MRI in patients with IDHmt and 1p/19q-codeleted oligodendroglioma.^106,107^ Several studies have linked deletions on 9p encompassing the *CDKN2A/B* locus on 9p21 to shorter survival in patients with grade 3 IDHmt and 1p/19q-codeleted oligodendroglioma, although not invariably.^107,108^ Homozygous deletion of *CDKN2A* has been found in approximately 10% of grade 3 oligodendroglioma, IDH-mutant, and 1p/19q-codeleted, but was not detected in grade 2 tumors (Table 4).^28,108^ Tumors with homozygous *CDKN2A/B* deletion typically demonstrate contrast enhancement upon imaging, and within grade 3 IDH-mutant and 1p/19q-codeleted oligodendroglioma, patients with tumors with a homozygous *CDKN2A* deletion had shorter survival.^28,50,91^ In addition, *CDKN2A/B* homozygous deletions are frequent in the recently identified aggressive oligosarcoma subgroup.^109^ Point mutations in *CDKN2A* or *CDKN2B,* amplification of *CDK4* or *CCND1*, and homozygous deletion of *RB1* are rare or absent in oligodendroglioma, IDH-mutant and 1p/19q-codeleted (Table 4), and so far no prognostic roles for *CDK4* amplification and homozygous deletion of *RB1* in grade 3 IDHmt and 1p/19q-codeleted oligodendroglioma patients has been identified.^28^

**Table 4.** The Frequency of Alterations in IDH-Mutant and 1p/19q-Codeleted Oligodendroglioma Grades 2 and 3

Gene	Dataset	Grade 2	grade 3
*CDKN2A* homodel	TCGA	0/48 (0%)	0/37 (0%)
	MSK	0/36 (0%)	0/43 (0%)
	POLA		33/483 (7%)
CIC	TCGA	27/48 (56%)	20/37 (54%)
	MSK	25/36 (69%)	34/43 (79%)
FUBP1	TCGA	12/48 (35%)	11/37 (29%)
	MSK	8/36 (22%)	16/43 (37%)
NOTCH1	TCGA	4/48 (8%)	14/37 (38%)
	MSK	2/36 (5%)	14/43 (32%)
PIK3CA	TCGA	10/48 (21%)	6/37 (16%)
	MSK	3/36 (8%)	14/43 (32%)
PIK3R1	TCGA	2/48 (4%)	4/37 (11%)
	MSK	5/36 (14%)	7/43 (16%)
CDK4ampl	POLA		1/483

TCGA: data extracted from Gliovis.

MSK: data extracted from https://www.cbioportal.org/study/summary?id=msk_impact_2017.

POLA: reference^89^.

### CIC and FUBP Mutations

Up to 70% of IDH-mutant and 1p/19q-codeleted oligodendroglioma carry inactivating mutations in the homolog of the *Drosophila* capicua gene (*CIC*) on 19q13.2.^110,111^*CIC* inactivation has been shown to cooperate with mutant IDH in fostering increased production of 2-hydroxyglutatrate (2-HG), and has been linked to aberrant activation of MAPK/Ras signaling.^112,113^ Despite some studies reporting an association between *CIC* mutation or loss of protein expression and outcome, most studies in patients with grade 2 or 3 IDH-mutant and 1p/19q-codeleted oligodendroglioma found no prognostic association of *CIC* mutations.^20,108,114,115^ Although *FUBP1* is frequently mutated in IDH-mutant and 1p/19q-codeleted oligodendroglioma, there is no clear association with survival.^108^ This is despite the mutation being subclonal (similar to CIC mutations) which suggests selection for *FUBP1* mutant clones during tumor evolution.

### Other Genetic Alterations

A few additional genetic alterations present in minor subsets of oligodendroglioma, IDH-mutant, and 1p/19q-codeleted each have been linked to unfavorable prognosis. These include mutations in *PIK3CA* and *NOTCH1* (Table 4).^20,116,117^ The rare absence of *TERT* promoter mutations in IDH-mutant and 1p/19q-codeleted oligodendroglioma (occurring in < 5% of cases) has been associated with a worse prognosis in one study.^118^ In addition, increased MYC signaling has been found in a clinically more aggressive subgroup of IDHmt and 1p/19q-codeleted oligodendroglioma demonstrating an oligodendrocyte precursor-like gene expression signature, and *MYC* gain has been shown to be associated with the risk of developing a post-TMZ hypermutated phenotype.^119,120^*PTEN* alterations have been associated with shorter survival of patients with grade 2 IDHmt and 1p/19q-codeleted oligodendroglioma in one study.^108^

### Chromosomal Instability and Tumor Mutational Burden in IDH-Mutant 1p/19q Codeleted Oligodendroglioma

Several retrospective studies reported that 1q and 19p polysomy is detectable in subsets of IDH-mutant and 1p/19q-codeleted oligodendroglioma and is associated with earlier recurrence and shorter survival.^121^ Chromosomal copy number variations in addition to 1p/19q codeletion increase significantly from grades 2 to 3 in IDH-mutant and 1p/19q-codeleted oligodendroglioma.^20,122^ In addition to a distinct DNA methylome profile, the recently reported prognostically unfavorable oligosarcoma also features increased chromosomal copy number variations.^109^ Apart from this rare subgroup, studies have reported that copy number burden was associated with a less favorable outcome in patients with IDH-mutant and 1p/19q-codeleted oligodendroglioma.^20,123^ In addition, a study based on TCGA data sets from 169 IDHmt and 1p/19q-codeleted oligodendroglioma patients revealed that high tumor mutational burden, defined by ≥ 0.69 mutations/megabase of DNA, was significantly associated with shorter survival.^124^ This study found a similar trend when using GLASS data sets from a small cohort of 25 IDHmt and 1p/19q-codeleted oligodendroglioma patients. Treatment with temozolomide has been associated with recurrences with high tumor mutational burden, high grade, and contrast-enhancing recurrences with poor prognosis.^57,59^

### Gene Expression Profiles

Microarray-based mRNA expression profiling of 68 IDH-mutant and 1p/19q-codeleted oligodendroglioma treated with radio- +/− chemotherapy revealed an 8-gene signature (*ST3GAL6, QPCT, NQO1, EPHX1, CST3, S100A8, CHI3L1*, and *OSBPL3*) whose overexpression was significantly associated with shorter PFS.^81,125^ Another study reported on a prognostic 35-gene signature that identified high-risk and low-risk subgroups of 1p/19q codeleted glioma patients.^126^ A more recent study based on TCGA datasets from 137 oligodendroglioma patients and 2 independent validation cohorts of 218 patients reported on gene expression-based distinction of 2 prognostically distinct subtypes of oligodendroglioma, IDH-mutant and 1p/19q-codeleted.^127^ The prognostically unfavorable subtype displayed a proliferative phenotype with enrichment of histologically grade 3 tumors and higher mutation frequency in *EGFR*, *MET,* and *NOTCH1*. Using integrated analysis of the transcriptome, genome, and methylome data from 156 IDHmt and 1p/19q-codeleted oligodendroglioma patients, 3 subgroups were identified with distinct gene expression patterns corresponding to oligodendrocyte, oligodendrocyte precursor cell, and neuronal lineage cells.^120^ Among these, the oligodendrocyte precursor cell-like subgroup showed aberrant MYC activation and significantly worse outcomes independently of histological grade.

## Methylation Analysis of IDH-Mutant Gliomas

Recent advances in glioma research underscore the pivotal role of epigenomic characteristics, particularly DNA methylation, allowing for refinement of the classification of gliomas, in particular in n IDH-mutant astrocytomas.^128^ Within the IDH-mutant astrocytoma tumor subgroup, 2 major methylation subgroups have been identified by genome-wide DNA methylation profiling: Glioma-CpG Island Methylator Phenotype (G-CIMP)-low and G-CIMP-high, with G-CIMP-low tumors displaying lower levels of genome-wide DNA-methylation levels than G-CIMP-high tumors.^98^ Patients with an initial diagnosis of G-CIMP-low tumors exhibited shorter overall survival compared to those with G-CIMP-high, accompanied by notable alterations in cell cycle pathways, *CDKN2A/B* deletions, and *MET* amplifications.^90,98,104,129^ The recently described LINE-1 methylation sequencing can be used as proxy for genome-wide DNA-methylation levels of a sample, and samples with low LINE-1 methylation levels are associated with grade 4 histology.^29^ Longitudinal analyses of paired samples showed that some G-CIMP-high tumors transitioned to G-CIMP-low at the time of tumor progression, indicating genome-wide DNA- demethylation.^130,131^ Tumors without malignant progression did not show such large differences, and a third methylation profile “G-CIMP-high at risk to low” subsequently identified patients with an intermediate prognosis.^132,133^ In parallel to these findings, a German group developed a classifier to aid the typing of brain tumors.^134^ This “Heidelberg methylation classifier” distinguishes an “Astrocytoma, IDH-mutant; high grade” class (A IDH HG) from an “Astrocytoma, IDH-mutant; lower grade” (A IDH) class, with different clinical outcomes.^135^ The prognostic significance of this distinction was confirmed in independent datasets, both in in grade 3 and in grades 2–4 astrocytomas.^24,73^ Multivariate analysis including grade, homozygous deletion of *CDKN2A/B* and methylation class showed that the latter 2 were independent prognostic factors, but not histological grade.^73^ Hypermethylation of a set of 7 HOX genes has been associated with survival in both IDH-mutant astrocytoma and IDH-mutant and 1p/19q codeleted oligodendroglioma, independent from CIMP status.^104^

MGMT promoter methylation is frequent in IDH-mutant glioma, and is present in nearly all oligodendrogliomas IDHmt and 1p/19q-codeleted.^136^ In grade 4 astrocytoma IDHmt, MGMT methylation status was associated with improved outcome but in the CATNON trial on anaplastic astrocytoma IDHmt, it failed to predict outcome to the addition of temozolomide to radiotherapy.^137,138^ In a study comparing radiotherapy to temozolomide chemotherapy in grade 2 gliomas MGMT status appeared to be associated with a longer PFS in astrocytoma IDHmt after temozolomide treatment, but whether MGMT status has an effect on survival in grade 2 astrocytoma IDHmt is unclear.^139^ In IDH-mutant low-grade gliomas, the MGMT methylation level in the tumor at first resection was associated with a hypermutational status at tumor recurrence after temozolomide treatment.^140^ To conclude, MGMT promoter assessment is not useful in oligodendroglioma IDHmt and 1p/19q codeleted, its clinical value in IDHmt astrocytoma remains to be demonstrated and is most likely less relevant than CIMP status.

IDH-mutant, 1p/19q codeleted oligodendrogliomas represent a distinct methylation class, and to date, methylation analysis with respect to tumor grade or along the disease course is not well understood. A recently identified and prognostic unfavorable methylation subclass “oligosarcoma” contains a high proportion of IDH-mutant and 1p19q codeleted oligodendrogliomas.^109^ Several of these “oligosarcomas” were recurrences of prior oligodendrogliomas which suggests this subclass represents malignant progression of oligodendrogliomas.

Overall, the analyses to date lead to biological insights that shed light on the role of global DNA methylation changes and demethylation in specific genes during glioma progression. While the utility of these classifiers and biomarkers in treatment strategies awaits further validation, their emergence marks a significant step towards personalized and risk-adapted approaches in the management of gliomas.

## Clinical Experience With IDH Inhibitors in Grades 2 and 3 IDH-Mutant Glioma Patients

The importance of IDH mutations in gliomagenesis has made it a target of interest for the treatment of IDH-mutant gliomas.^141,142^ Multiple IDH inhibitors have been developed, some specific for mutant IDH1, while others inhibit both mutant IDH1 and IDH2. These inhibitors have been evaluated in several studies with heterogeneous populations of patients with IDH-mutant grades 2–4 gliomas, both in patients with enhancing and non-enhancing tumors (Table 5).^4–6,143,144^ All the initial studies of IDH inhibitors were conducted in patients with recurrent/progressive gliomas where the histology was usually based on prior resections rather than on tumor samples obtained immediately before study entry.^5,6,143–146^

**Table 5.** Summary of the various clinical studies on IDH inhibitors, the objective response rate and the median progression-free survival in enhancing and unenhancing glioma.

Study	IDH inhibitor	Patient population investigated	Number of patients included	Glioma types/grades	ORR and mPFS in enhancing gliomas	ORR and mPFS in non-enhancing gliomas
Mellinghoff et al^133^	Ivosidenib	Recurrent IDH1-mutant gliomas	66: 31 enhancing 35 non-enhancing	32 grade 2 18 grade 3 12 grade 4	0 (0%) 14 SD (45.2%) mPFS 1.4 m	2.9% (1 PR) 30 SD (85.7%) mPFS 13.6 m
Mellinghoff et al^5^	Vorasidenib	Recurrent IDH1/2 mutant gliomas	52: 30 enhancing 22 non-enhancing	25 grade 2 22 grade 3 4 grade 4	0 (0%) 17 SD (56.7%) mPFS 3.0 m for grade 2; 3.7 m for grade 3	18% 1 PR (4.5%) 3 MR (13.6%) 16 SD (72.7%) mPFS 19.6 m for grade 2; 40.8 m for grade 3
Mellinghoff et al^131^	Vorasidenib and Ivosidenib	Recurrent, non-enhancing IDH1-mutant gliomas	49, all non-enhancing	43 grade 2 6 grade 3	N/A	Vorasidenib 50 mg qd: 42.9% (2 PR, 4 MR); SD 42.9% Vorasidenib 10 mg qd: 10% (1 MR); SD 80% Ivosidenib 500 mg qd: 35.7% (3 PR, 2 MR); SD 64.3% Ivosidenib 250 mg BID: 12.5% (1 MR); SD 62.5%
Mellinghoff et al^7^	Vorasidenib	Residual or recurrent non-enhancing gliomas	168, all non-enhancing	Grade 2	N/A	
Natsume et al^6^	DS-1001	Recurrent IDH1-mutant gliomas	47: 35 enhancing 12 non-enhancing	16 grade 2 22 grade 3 9 grade 4	17.1% (2 CR, 4 PR) 11 SD (31.4%) mPFS 10.4 weeks	33.3% (1 PR, 3 MR) 7 SD (58.3%) mPFS not reached
De la Fuente et al^132^	Olutasidenib	Recurrent IDH1-mutant gliomas	26: 23 enhancing 3 non-enhancing	4 grade 2 15 grade 3 7 grade 4	8.7% (2 PR) 10 SD (38.5%) in the entire cohort	0% 10 SD (38.5%) in the entire cohort
Wick et al^134^	BAY1436032	Recurrent IDH1-mutant gliomas	49: 35 LGG, 33 of whom had measurable enhancing disease 14 enhancing grade 4 astrocytoma		LGG: 6.1% (1 CR, 1 PR) 15 SD (42.9%) Grade 4 astrocytoma: 0% 4 SD (29%)	LGG: 100% (2 PR)

IDH, isocitrate dehydrogenase; LGG, low-grade glioma; mPFS, median progression-free survival; MR, minor response; ORR, objective response rate; PR, partial response; SD, stable disease.

Ivosidenib, the first-in-class IDH 1 inhibitor, was evaluated in a multicenter, open-label, phase 1 study in 66 patients with recurrent IDH1-mutant gliomas, 32 of whom had grade 2 gliomas, 18 had grade 3 gliomas, and 12 had grade 4 gliomas.^145^ Only one partial response was observed, in a patient with grade 3 a non-enhancing tumor. Stable disease as the best response was seen in 85.7% of patients with non-enhancing tumors compared to 45.2% with enhancing tumors. Disease progression was more prevalent in enhancing tumors (54.8% vs. 11.4%), and median PFS was longer in non-enhancing tumors (13.6 months) versus enhancing tumors (1.4 months). More recent analysis of data from the ivosidenib trial by tumor grade showed no objective responses, neither in enhancing nor in non-enhancing tumors.^4^ For patients treated at the dose expansion cohort with non-enhancing tumors the median PFS was 19.4 months for grade 2 tumors (18 patients) and 23 months for grade 3 gliomas (4 patients; data on file). These data suggest that some patients with recurrent grade 3 non-enhancing gliomas may benefit from ivosidenib.

Another phase 1 study evaluated the brain-penetrant IDH1/2 inhibitor vorasidenib in recurrent IDH1/2-mutant gliomas. Seventeen of the twenty-five grade 2 gliomas, 5 of the 22 grade 3 gliomas, and none of the 4 grade 4 gliomas were non-enhancing (data on file).^5^ The objective response rate (ORR) was 18% in non-enhancing gliomas (1 partial response and 3 minor responses), and 3.3% in enhancing gliomas (1 patient with a grade 3 tumor). The ORR was 8% for grade 2 gliomas and 13.6% for grade 3 gliomas. Median PFS was again longer in patients with non-enhancing tumors (36.8 vs. 3.6 months). Of the 22 non-enhancing tumors, there were 17 grade 2, 5 grade 3, and no grade 4. Median PFS was 19.6 months for grade 2 tumors and 40.8 months for grade 3 tumors. There were 30 patients with enhancing tumors, 8 grade 2, 17 grade 3, and 4 grade 4 (1 grade unknown). Median PFS was 3.0 months for grade 2, 3.7 months for grade 3, and 1.1 months for grade 4 gliomas. These data, while limited, suggest that the response to vorasidenib is not substantially different between grades 2 and 3 gliomas, especially when they are non-enhancing.

Ivosidenib and vorasidenib were both evaluated in a surgical window-of-opportunity trial in patients with recurrent non-enhancing grade 2 (43 patients) and 3 (6 patients) IDH1-mt gliomas.^143^ Two doses of vorasidenib and ivosidenib were used. Five patients had a partial response, and 8 patients had a minor response. Most responders had grade 2 tumors, although one patient with grade 3 astrocytoma had a partial response. Stable disease was documented in 31 patients. Vorasidenib was associated with a slightly tighter reduction in intra-tumoral 2-HG levels and was selected for evaluation in the phase 3 placebo-controlled INDIGO study. This trial enrolled patients with grade 2 IDH-mutant tumors with measurable non-enhancing disease, not in immediate need of chemotherapy/radiation, which study found that vorasidenib led to significant improvement in PFS and time-to-next intervention compared to placebo.^7^

A multicenter, open-label, dose-escalation, phase 1 study evaluated the IDH1 inhibitor DS-1001 (safusidenib, AB-218) in patients with recurrent IDH-mutant gliomas of any grade.^6^ Of the 45 patients, 4 had grade 2 oligodendroglioma, 11 had grade 3 oligodendroglioma, 12 had grade 2 astrocytoma, 11 had grade 3 astrocytoma, and 7 had grade 4 astrocytoma. Thirty-five patients had enhancing tumors and 12 had non-enhancing tumors. ORR was 33.3% in non-enhancing tumors and 17.1% in enhancing gliomas.^6^ A complete response was observed in an enhancing grade 4 astrocytoma and an enhancing grade 3 oligodendroglioma, and partial responses were observed in enhancing astrocytomas (2 of the 21) and oligodendrogliomas (2 of the 9). Stable disease was reported in 52.3% of enhancing astrocytomas and 22.2% of enhancing oligodendrogliomas. Median PFS was longer in non-enhancing tumors (not reached) versus 10.4 weeks in enhancing tumors. Although the paper did not breakdown the data by tumor grade, since the majority of patients had grade 3 tumors, DS-1001 (safusidenib) appears to be active for both enhancing and non-enhancing grade 2 and 3 IDH-mutant gliomas.

Another multicenter phase 1 study evaluated BAY1436032 in 29 patients with recurrent IDH-1 mutant gliomas.^146^ There was a complete response in one grade 3 astrocytoma patient, and a partial response in a grade 3 astrocytoma and 2 grade 3 oligodendroglioma patients. In this study, 33 of the 35 patients with grades 2 and 3 glioma had enhancing disease, among whom 1 had a complete response, and another had a partial response, while 2 had stable disease. Twenty-nine percent of grade 4 astrocytoma patients had stable disease, but no responses were observed among these patients. PFS rate at 3 months was higher in grades 2 or 3 IDH-mutant gliomas compared to grade 4 IDH-mutant astrocytomas patients (31% vs. 22%).

Finally, the IDH1 inhibitor olutasidenib was evaluated in a phase Ib/II study, in 26 patients with recurrent IDH-1 mutant gliomas of whom 88% had enhancing tumors.^144^ Fifty-eight percent of the patients had a grade 3 glioma, and 27% had a grade 4 astrocytoma. A partial response was observed in 2 patients, who both had enhancing gliomas (1 grade 3 and 1 grade 4). Upon central assessment of response, 4 partial responses were reported, and 5 patients had a reduction in tumor size that did not reach 50%. PFS rate at 6 months was 23% in enhancing gliomas.

All patients with enhancing and higher-grade gliomas included in the above studies had recurrent diseases and usually had received multiple lines of treatment prior to enrollment. Prior radiation was given to 73% to 100%, and prior chemotherapy to 75% to 88% of the enrolled patients, with most patients having received more than 2 prior lines of therapy.^5,6,144–146^ At advanced stages, tumors can acquire additional genomic alterations that can bypass the need for the mutant IDH enzyme to maintain gliomagenesis and the IDH mutation can even be lost due to copy number alterations.^97,142^ Despite this, a number of patients with grade 3 IDH-mutant gliomas appear to have benefited from therapy with IDH inhibitors, challenging the perception that IDH inhibitors would be efficacious only in grade 2 non-enhancing IDH-mutant gliomas. It is possible that the benefit of IDH inhibitors will be even greater in newly diagnosed tumors grade 3 IDH-mutant gliomas where activation of alternate molecular drivers may be less than in recurrent disease.

## Conclusions

While some series show an association between tumor grade and outcome others fail to do so, and many patients with a grade 3 IDH-mutant astrocytoma or IDH-mutant and 1p/19q codeleted oligodendroglioma experience a survival well beyond 10 years. The wide overlap in the survival range of grades 2 and 3 IDH-mutant glioma patients appears in part due to the subjective nature of the histological grading of IDH-mutant tumors: There are no sharp and objective criteria distinguishing grade 2 form grade 3 tumors. Nonetheless, many factors associated with the outcome of patients with IDH-mutant glioma (enhancement on imaging, molecular findings) show some association with histological grade. Still, the overall limited association of outcome of patients with grade 2 versus 3 3 IDH-mutant glioma patients reflects the biological continuum of these tumor grades implying that a sharp distinction between these grades is artificial.

Genetic analysis analyses may allow for better prognostication of IDH-mutant glioma patients than histological grading and mitotic counts. An update of the WHO classification especially for IDHmt astrocytomas is needed. Apart from homozygous deletion of *CDKN2A/B* several other well-defined alterations have been associated with poor outcome. In contrast, there are currently no well-validated prognostic molecular markers in IDH-mutant and 1p/19q-codeleted oligodendroglioma patients. Similar to mutational analysis, genome-wide methylation analysis holds promise for risk stratification. Several studies have shown that specific methylation patterns are associated with outcome, may allow risk stratification within tumor grades, and may be more associated with outcome than tumor grade.

The modest association between age and outcome of patients with IDH-mutant glioma does not warrant the use of guidelines of a strict age criterion of 40 years for the identification of patients at risk for a poor outcome. The available evidence shows that a cutoff in the range of 50-60 years is more appropriate for that purpose. Importantly, most series show that the prognostic effect of clinical factors decreases once the extent of resection and in particular the quantitative postoperative tumor volume are considered. This is in particular true for patients with IDH-mutant astrocytoma, in whom postoperative residual tumor has a major association with survival.

Although the presence of contrast enhancement on MR imaging is associated with tumor grade in IDH-mutant gliomas, this finding is neither sensitive or specific, and a substantial percentage of grade 2 oligodendrogliomas and IDH-mutant astrocytomas will demonstrate some level of enhancement. Contrast enhancement at the time of progression of lower-grade IDH-mutant gliomas suggests transformation towards a higher grade of malignancy, which carries a different clinical significance compared to the presence of contrast enhancement at first diagnosis. For prognostication, spontaneous TGR may help in predicting the clinical behavior of IDH-mutant glioma. The evaluation of changes in tumor growth curves may be a better way of identifying patients with non-enhancing tumors that benefit from medical treatments than classical response assessment using RANO criteria, and needs further confirmatory studies. Clinical trials with IDH inhibitors have shown some activity in recurrent tumors, in enhancing tumors, and in grade 3 tumors. From a biological perspective, there is no a priori reason why newly diagnosed grade 3 tumors cannot be responsive to IDH inhibitors and other clinical, radiological, and molecular factors need consideration (like growth rate, histology, other molecular findings, and pre-and postoperative tumor volume). Real-world studies should prospectively collect data to provide guidance for future patient counseling.
