# Erratum to: The biological significance of tumor grade, age, enhancement, and extent of resection in IDH-mutant gliomas: How should they inform treatment decisions in the era of IDH inhibitors?

**DOI:** 10.1093/neuonc/noae185

**Published:** 2024-09-14

This is an erratum to: Martin J van den Bent, Pim J French, Daniel Brat, Joerg C Tonn, Mehdi Touat, Benjamin M Ellingson, Robert J Young, Johan Pallud, Andreas von Deimling, Felix Sahm, Dominique Figarella Branger, Raymond Y Huang, Michael Weller, Ingo K Mellinghoff, Tim F Cloughesy, Jason T Huse, Kenneth Aldape, Guido Reifenberger, Gilbert Youssef, Philipp Karschnia, Houtan Noushmehr, Katherine B Peters, Francois Ducray, Matthias Preusser, Patrick Y Wen, The biological significance of tumor grade, age, enhancement, and extent of resection in IDH-mutant gliomas: How should they inform treatment decisions in the era of IDH inhibitors?, *Neuro-Oncology*, 2024;, noae107, https://doi.org/10.1093/neuonc/noae107

In the originally published version of this manuscript, a typographical error was present in the name of author Tim F. Cloughesy.

This has been corrected.

